# Mesothelioma incidence surveillance systems and claims for workers’ compensation. Epidemiological evidence and prospects for an integrated framework

**DOI:** 10.1186/1471-2458-12-314

**Published:** 2012-07-05

**Authors:** Alessandro Marinaccio, Alberto Scarselli, Enzo Merler, Sergio Iavicoli

**Affiliations:** 1 Occupational Medicine Department, INAIL (Italian Workers Compensation Authority) research area, Italian National Mesothelioma Register (ReNaM), Via Alessandria 220, Rome, 00198, Italy; 2 Mesothelioma Register of the Veneto Region, Padua Local Health Unit, Via dell’Ospedale 22, Padua, 35128, Italy

## Abstract

**Background:**

Malignant mesothelioma is an aggressive and lethal tumour strongly associated with exposure to asbestos (mainly occupational). In Italy a large proportion of workers are protected from occupational diseases by public insurance and an epidemiological surveillance system for incident mesothelioma cases.

**Methods:**

We set up an individual linkage between the Italian national mesothelioma register (ReNaM) and the Italian workers’ compensation authority (INAIL) archives. Logistic regression models were used to identify and test explanatory variables.

**Results:**

We extracted 3270 mesothelioma cases with occupational origins from the ReNaM, matching them with 1625 subjects in INAIL (49.7%); 91.2% (1,482) of the claims received compensation. The risk of not seeking compensation is significantly higher for women and the elderly. Claims have increased significantly in recent years and there is a clear geographical gradient (northern and more developed regions having higher claims rates). The highest rates of compensation claims were after work known to involve asbestos.

**Conclusions:**

Our data illustrate the importance of documentation and dissemination of all asbestos exposure modalities. Strategies focused on structural and systematic interaction between epidemiological surveillance and insurance systems are needed.

## Background

Malignant mesothelioma (MM) is a rare and rapidly fatal tumour closely related to inhalation of asbestos fibres. It arises from the serous membranes of the pleura and, less frequently, from the peritoneal and pericardial cavities and the tunica vaginalis testis. Many western countries are currently suffering an MM epidemic, reflecting the widespread use of asbestos between the 1950’s and 80’s in many industrial applications.

In Italy, from the end of the Second World War to the asbestos ban in 1992, 3,748,550 tons of raw asbestos were produced, with a peak between 1976 and 1980, of more than 160,000 tons/year [1]. While asbestos consumption levelled off during the 1960s and 1970s in the United States, Australia, United Kingdom and Nordic countries, it did not do so until the early 1980s in Italy and France [2]. Forecasts of the incidence of MM and mortality trends in various countries are strongly influenced by asbestos consumption patterns in the past [3-7].

The Italian system of compensation for occupational diseases was established in 1926 and works on the basis of a list of occupational diseases and exposures. The system applies principles similar to those defined around the same time in other European countries, such as France, UK, and Germany [8]. In 1994 mesothelioma (of every site) was recognized for the first time in Italy as an occupational disease, in addition to lung cancer (every type) occurring in asbestos exposed workers. The Italian Workers’ Compensation Authority (INAIL) allows compensation in response to individual workers’ claims, and benefits are granted to the subject or to relatives.

Mesothelioma is recognized as an occupational disease, but environmental (living in the neighborhood of an industrial or natural source of asbestos) or indirect exposures (living with a person occupationally exposed to asbestos) have also been associated with it [9].

A permanent MM epidemiologic surveillance system, based on a national register (ReNaM), has been established in Italy to estimate incidence rates, investigate asbestos exposures, identify any possible underestimated or unknown sources of asbestos contamination, and promote research and reclamation programs. ReNaM has published figures for incidence, survival, latency and asbestos exposure [10-12]. Recently ReNaM reported a case list of 9,166 MM cases diagnosed from 1993 to 2004; the modalities of exposure were investigated for 6,640 of these, and occupational exposure was found in 69.9% (81.9% in men and 33.4% in women) [13].

The aim of this study was to examine the rates of claims filed and compensations awarded, and the demographic, social, geographic, diagnostic, etiologic and professional factors associated with the probability of seeking and receiving compensation. Identification of such factors may inform policies around communication to workers and clinicians regarding MM, and lead to improved efficiencies in the public insurance system.

## Methods

To estimate the MM incident cases seeking or not seeking and receiving or not receiving compensation, we implemented an individual linkage between the ReNaM and INAIL archives. ReNaM is an epidemiological surveillance and research system with a regional structure: operating centres (COR) have gradually been set up in 18 of the 20 Italian regions, covering almost the whole country (98.5% of the Italian population). CORs actively search for incident cases and investigate occupational and residential history, and lifestyle habits using a standardized questionnaire administered by a trained interviewer to subjects or next of kin. Occupational exposure is classified qualitatively, on the basis of questionnaire, as definite, probable or possible considering the probability, intensity and duration of exposure. More details are available in the national guidelines regarding diagnostic and anamnesis classification criteria [14]. Cases are assigned to specific economic sectors (38 categories) considering the whole occupational history. We further classified them into four macro-groups, according to the modalities of asbestos use: *direct use* including activities with use of asbestos as “material” (shipbuilding and repair, asbestos-cement industry, railway rolling stock construction and maintenance, asbestos mining, port handling, asbestos textile industry, friction materials production, production of gaskets and packaging); *indirect use for insulation and auxiliary tools* (metal and engineering, metallurgic, oil refineries, metal, food and drink industries, sugar refineries, organic and inorganic chemical plants, wood processing, tobacco, leather tanning, non-asbestos textile finishing, glass and ceramic, paper, jewellery, gas production, navy and military defense, power plants, heat and steam generators); *construction sector* (the whole building industry); *atypical exposures* including workers for which there is a lack of information about asbestos exposure that results frequently unexpected (car mechanics, jute sack recycling, agriculture, fishery, bars and restaurants, public administration, education, banks, post offices, health and social services).

From the ReNaM archives we selected MM cases diagnosed in the period 2000–2004. The regions of Valle d’Aosta, Piedmont, Veneto, Lombardy, Friuli-Venezia Giulia, Tuscany, Liguria, Emilia-Romagna, Marche, Apulia, Basilicata, Campania, and Sicily (77.7% of the whole Italian population) provided incidence data; caselists from Lazio, Abruzzo, Calabria and Sardinia (17.5%) were not considered complete and Trentino Alto Adige, Umbria and Molise (4.8%) did not transmit data for the selected period. Only cases with pleural, peritoneal, or pericardial forms and with occupational exposure (definite, probable or possible) were considered.

INAIL receives a worker’s compensation claim for mesothelioma and verifies the diagnosis. The claim can be rejected if there was no occupational exposure to asbestos, or the worker was not insured by INAIL, or for inadequate documentation. We selected all mesothelioma claims from the national workers’ compensation authority (INAIL) registered in the period 2000‐2007 (to include dossiers with delayed definition) from the whole country (to include people with residence and workplace in different regions). The linkage was conducted using personal identification numbers (tax numbers) or a combination of full name, sex, date and place of birth.

Logistic regression models were used to determine the relative risk of not making a claim for the mesothelioma incident cases eligible for compensation, and their demographic, geographic and occupational variables. We calculated the level of significance and its 95% confidence limits. Reference categories in the logistic model were chosen with respect to the exposure modalities with the highest percentage of claims for each variable (sex, age at diagnosis, year and certainty of diagnosis, region of residence/work, level of exposure and occupational category).

Individual data have been made available for research studies by ReNaM and by INAIL and have been used strictly only to perform linkage procedures. The data were analyzed with SAS statistical software (LOGISTIC procedure, SAS version 9.1; SAS Institute Inc., North Carolina), using the backward selection method in order to obtain the best-fitting model.

## Results

**Figure 1:**
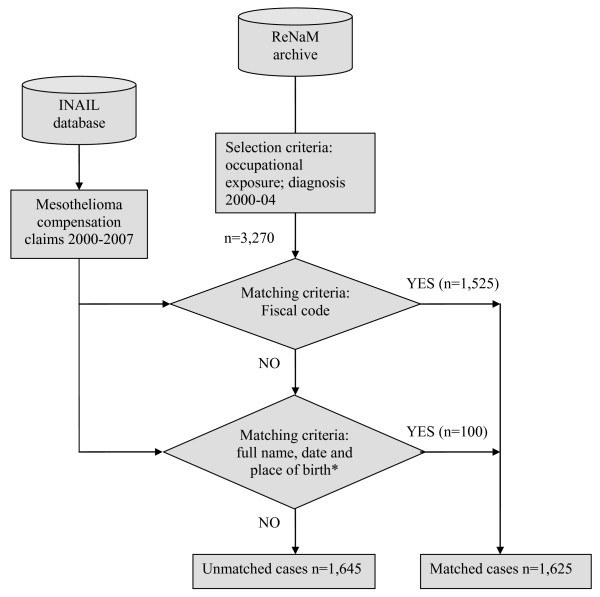
Flow chart of the mesothelioma cases selection and linkage procedures.

At present the ReNaM archives include 9,544 mesothelioma incident cases diagnosed from 1993 to 2004 and the modalities of exposure to asbestos have been investigated for 7,044 of them. Applying the selection criteria presented in Figure 1 (diagnosis between 2000 and 2004 and recognized occupational exposure), 3,270 MM cases were extracted from the ReNaM. Matching by personal tax numbers, we found 1,526 cases in the INAIL database; relaxing the criteria for linkage and searching by full name and place of birth, the number of matched subjects reached 1,625 (49.7%); 91.2% (1482) of these claims for MM received compensation from INAIL.

The proportion of cases with an occupational exposure identified by the Register who filed a compensation claim strongly reflected geographical, historical, demographic and etiologic factors. Only one-third of women with MM and with occupational exposure to asbestos filed for compensation; the proportion was more than double for men (Table 1). It decreased steeply with age at diagnosis. There were no significant differences for pleural or peritoneal forms and the proportions were close to the overall mean. There were too few pericardial cases to permit any meaningful conclusions.

**Table 1:** Malignant mesothelioma cases collected by the Italian national mesothelioma register (ReNaM) by sex, age classes, year of diagnosis, anatomical site, level of diagnostic certainty, area of residence, level of occupational exposure and category

	Compensation not claimed (n = 1645)		Compensation claimed (n = 1625)		
Variable	n	%	n	%	Total
					(n = 3270)
Gender					
Male	1355	47.78	1481	52.22	2836
Female	290	66.82	144	33.18	434
Age at diagnosis					
<45	14	42.42	19	57.58	33
45-54	111	39.08	173	60.92	284
55-64	363	41.11	520	58.89	883
65-74	600	50.63	585	49.37	1185
75+	557	62.94	328	37.06	885
Cancer site					
Pleura	1554	50.16	1544	49.84	3098
Peritoneum	84	51.85	78	48.15	162
Pericardium	7	70.00	3	30.00	10
Year of diagnosis					
2000	330	56.51	254	43.49	584
2001	328	49.62	333	50.38	661
2002	348	50.43	342	49.57	690
2003	342	49.85	344	50.15	686
2004	297	45.76	352	54.24	649
Type of diagnosis					
Certain	1264	47.13	1418	52.87	2682
Probable	210	55.70	167	44.30	377
Possible	171	81.04	40	18.96	211
Area of residence					
North-West	945	51.53	889	48.47	1834
North-East	278	37.57	462	62.43	740
Centre	141	44.34	177	55.66	318
South and Islands	281	74.34	97	25.66	378
Occupational exposure level					
Certain	874	39.14	1359	60.86	2233
Probable	267	67.59	128	32.41	395
Possible	504	78.50	138	21.50	642
Occupational category					
Direct use of asbestos	224	36.58	391	63.58	615
Presence of insulation	697	50.43	685	49.57	1382
Atypical exposure	424	58.37	309	42.16	733
Construction sector	300	55.56	240	44.44	540

Italy, 2000–2004, only cases with occupational exposure. Compensation claimed and not claimed.

**Figure 2:**
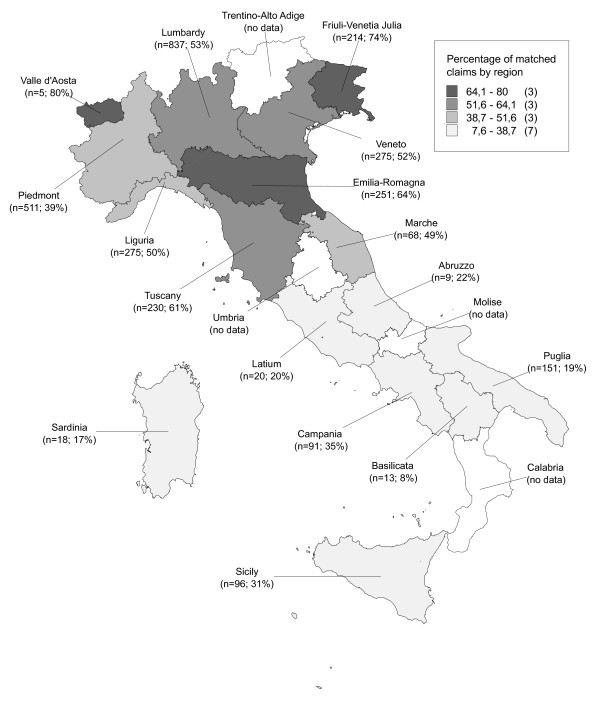
**Malignant mesothelioma cases with an occupational origin from national register.** Number and proportion of subjects seeking for compensation claim by Region (2000–2004).

The percentage of incident cases who filed claims for compensation rose almost steadily in the selected period, from 43.5% in 2000 to 54.2% in 2004. There was an evident North–south gradient, and the southern regions (islands included) had only about half the overall rate (25.7 vs. 49.7). More than 60% had definite occupational exposure, while only 32.4% and 21.5% respectively had probable and possible occupational exposure. Details by Region are reported in Figure 2.

Table 2 shows the results of the multiple logistic regression model for factors associated with compensation claims. Relative risk is the odds of not seeking compensation by individuals diagnosed with MM of occupational origin according to ReNaM epidemiological criteria. This risk, adjusted for all other variables included in the model, was significantly higher for women and for people older than 65 years. People living in Southern Italy had a risk of not seeking compensation more than four times that of north-eastern residents, and north-western patients had a significantly higher risk than the reference category (north-east).

**Table 2:** Logistic regression model

Variable	OR	95%	CI
Gender			
Male	1	Ref	
Female	1.734	1.347	2.231*
Age at diagnosis			
<45	1	Ref	
45-54	1.237	0.543	2.817
55-64	1.328	0.600	2.940
65-74	1.977	0.896	4.362
75+	3.124	1.406	2.142*
Year of diagnosis			
2000	1.667	1.297	2.142*
2001	1.138	0.893	1.451
2002	1.213	0.955	1.539
2003	1.139	0.896	1.447
2004	1	Ref	
Type of diagnosis			
Certain	1	Ref	
Probable	1.092	0.856	1.392
Possible	3.218	2.192	4.723*
Area of residence			
North-West	1.753	1.441	2.134*
North-East	1	Ref	
Centre	1.331	0.992	1.785
South and Islands	5.011	3.701	6.785*
Occupational exposure level			
Certain	1	Ref	
Probable	2.705	2.124	3.446*
Possible	4.564	3.660	5.692*
Occupational category			
Direct use of asbestos	1	Ref	
Presence of insulation	1.526	1.228	1.898*
Atypical exposure	1.705	1.321	2.199*
Construction sector	1.857	1.423	2.423*

Relative risk (and confidence interval at 95%) of not seeking compensation for MM cases with occupational origin, from Italian national mesothelioma register (ReNaM).

*Significant difference from reference category (Ref.) with 95% confidence interval.

Analyses by economic sector of exposure showed wide variability in the proportions of cases who filed for compensation. The highest rate was for railway workers involved in maintenance and repair or removing the asbestos insulation from rolling stock (73% of those seeking compensation). The proportion was 70.2% for port handling workers, 62.6% for shipbuilding and repair, 61.9% in the asbestos-cement industry, 62.7% in gas production and 61.1% in oil refineries. The construction sector had 44.4% of MM claims. After adjustment in the logistic model, the relative risk of not seeking compensation for construction workers reached 1.9 (95% CI 1.4-2.4), significantly higher than the reference category (people who used asbestos directly in the workplace). The lowest rates were for health and social services (32.3%), education (26.7%) and agriculture (8.6%).

**Figure 3:**
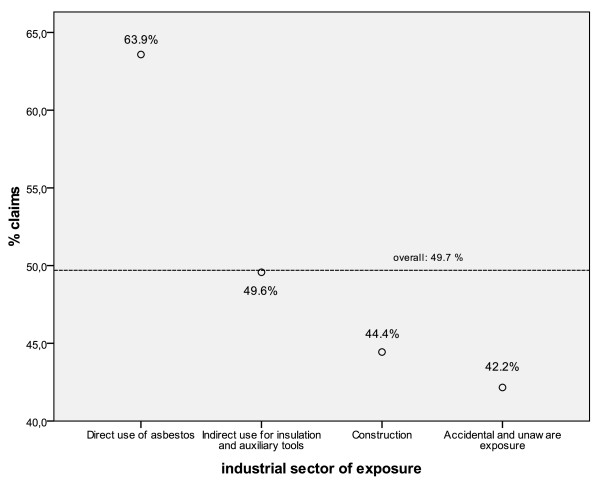
**Malignant mesothelioma cases with an occupational origin.** Proportion of subjects seeking for compensation claims by asbestos exposure modalities.

After grouping the four sectors by type of asbestos use, the proportions of cases seeking compensation and the adjusted relative risk of not seeking it are presented in Figure 3 and Table 2.

## Discussion

Comparisons of insurance compensation in relation to the epidemiological magnitude of occupational diseases are not frequently published despite the importance of this topic in evaluating inequalities and efficiencies of health care and welfare systems. The few published data regard MM because of the generally well known occupational etiology of the disease. For the 499 malignant mesothelioma cases recorded by the French Mesothelioma National Program in the period 1999‐2001, 309 (62%) sought recognition of an occupational disease, and it was granted for nearly all of them (91%). This proportion varies geographically and is closely related to physicians’ sensitivity to the occupational origin of the disease [15]. In Australia the Central Cancer Registry recorded 3090 malignant mesothelioma cases between 1972 and 2004, and the Dust Diseases Board compensated approximately 60% of these in that period [16]. A detailed analysis for the Canadian province of British Columbia reported 33% of MM cases in the provincial registry linked at the individual level with accepted claims for the period 1970‐2005 [17]. The critical point of these analyses is the lack of definition of occupational asbestos exposure by the epidemiological surveillance systems.

In Italy the ReNaM has been active since 1993 (compulsory since 2002) with the aims of defining the incidence rates and investigating each subject’s individual occupational, residential and environmental history using a structured questionnaire. Some important limitations regarding the ReNaM dataset bear discussion. The Register has not been developed uniformly throughout the country. This is why the selected period for analysis was 2000‐2004, guaranteeing a large enough sample and adequate territorial incidence coverage. Regions differ in their assessments of asbestos exposure with respect to incident cases, depending on the resources available. While the national guidelines for the standardization of MM cases collected aim to correct this imbalance, there are still large gaps among the different regions. Furthermore this study looked only at incident cases, not incidence rates and the reference populations of ReNaM and of the insurance system are not the same.

The overall rate of compensation for mesothelioma claims in Italy due to occupational exposure to asbestos is 49.7%, and more than 90% of these are granted by the workers’ compensation authority. A substantial number of people who deserve compensation for MM do not seek it. The percentage (50.3%) is higher than in the Canadian survey but no real comparison can be made because that registry does not specify industry sectors and occupational categories.

Compensated cases increased by 38.6% in the period 2000–2004 whereas the increase in the ReNaM occupational caselist was only 11.1% (from 584 in 2000 to 649 in 2004), indicating that epidemiological patterns only partially explain compensation decisions, which are influenced by sensitization and awareness [18].

In Italy owners are obliged to insure their workers for injuries and occupational diseases. However, the self-employed have no such obligation. INAIL is the institute with the main role, but some categories such as the military and firemen (jobs involving asbestos exposure) come under other specific insurance systems. Eligibility criteria for compensation include diagnostic certainty and an asbestos exposure suffered in the work place.

The likelihood of an individual with mesothelioma due to occupation seeking and receiving compensation was gender-specific. Mesothelioma is more frequent in men because of the larger proportion of male workers in the industrial sectors “historically” with high asbestos exposure, such as shipbuilding and repair, railway rolling stock maintenance, the asbestos-cement and construction industries. Thus the disease is under-recognized as being of occupational origin in women. The proportion of women with MM who were occupationally exposed and are collected in the Register is 13.3% but only 8.9% of cases received compensation. Probably women need to be better informed about the causes of the disease in view of the absence of a threshold for its occurrence [19].

The probability of seeking compensation for MM cases with occupational asbestos exposure declined steeply in relation to age at diagnosis. The ReNaM database indicates that age at diagnosis and diagnostic certainty are correlated. The proportion with a not-definite diagnosis was significantly higher among cases older than 75 years. This might well be because there is a tendency to avoid the use of invasive diagnostic methods in elderly and suffering patients [11]. Often too, retired people are less aware of an exposure risk in their workplace many years earlier. Clinicians need to become more aware of the need to enquire not only about a patient’s current work status but also about the work history. For occupational tumours, and particularly MM, the long latency (generally around 40 years), the variety of occupations involved in exposure and the absence of a threshold, often make it difficult to identify the correct etiology [20].

In the multivariate model, after adjustment for all variables, the region of residence at diagnosis remained a significant source of variation in the probability of not seeking compensation for occupationally exposed patients. This is worth stressing as an opportunity to define policies to reduce this source of inequality. The sensitivity and awareness of health care system operators (clinicians particularly) was far from uniform over the whole country, and the level was particularly low in the south of Italy. There is therefore a pressing need to systematically spread information about the causes of MM and patients’ rights, also using the ReNaM network.

At present only Italy, France [15] and Australia [21] have specific epidemiologic surveillance systems for mesothelioma cases based on active searches and individual interviews to analyze the occupational history of each case. Mesothelioma mortality surveillance is based on death certificates in Great Britain [22] and the United States [4], and also on territorial cancer registries in the United States [23] and Germany [24]. In the Nordic countries the complete development of national cancer incidence registries allows systematic linkage with occupational information archives [25-27].

Analyses to verify the extent of compensation for MM cases by economic sector of exposure are not frequently published. The highest rates of compensated cases are after occupational exposure in activities known to involve asbestos. Heavy asbestos exposure during the maintenance and disposal of insulation from railway carriages is often reported in Italy [28,29] and elsewhere [21,30-32]. The asbestos-cement industry [33,34] and shipbuilding and repair [35] provide the most detailed published studies on account of the number of plants involved and exposed workers in Italy. Our findings confirm that workers in these sectors and clinicians are well informed about the occupational origin of the disease. In contrast, where the worker has been exposed to asbestos during work but this was not evident, the probability of seeking (and receiving) compensation is much smaller. The MM cases due to exposure in the education sector, like in the social and health care services, provide evidence of this.

At present the most important area of exposure for MM cases collected by the Italian surveillance program is the construction industry, where asbestos has been used for fireproofing and acoustic insulation, in mixtures with cement and plastic and in vinyl flooring. As a result, construction workers could be involved in the risk of asbestos exposure during maintenance and restructuring activities [36]. The circumstances of asbestos exposure in the construction sector are generally not evident and not easy to define correctly, especially when no direct interview with the person concerned is available. A large proportion of MM cases exposed during work in this sector do not apply for compensation. Almost all construction workers are men, and in the adjusted analyses their relative risk was significantly elevated.

Our data highlight the importance of the documentation and dissemination of all asbestos exposure modalities since many - considering the large-scale use of asbestos and the absence of a threshold for the dose–response curve - are frequently not expected. Regulatory and public health agencies need effective notification systems to ensure that all individuals newly diagnosed with MM seek compensation benefits.

## Conclusions

The substantial proportion of workers with tumours of occupational origin who do not seek compensation and the consequent underestimation of the occupational cancer burden in insurance statistics is a public health topic [37]. Individually, many patients do not collect the compensation to which they are entitled, largely due to lack of information. Collectively, correct evaluation of the distribution of risk is fundamental for establishing insurance premiums, and correct premiums are fundamental to induce preventive measures for risk reduction. Furthermore, the health care costs of occupational tumours not known to the insurance system are shifted onto general public finance through the national health service, distorting its economics.

Asbestos exposure is a legacy of the past, but current exposure to carcinogens in the workplace is still an open issue. For MM, epidemiological analysis of the gap between compensation data and surveillance system findings is now possible in Italy and in other countries that have both these data sources [38]. For other tumour sites it is still hard to do this with what national data there are. The development of a complete occupational cancer surveillance system is a challenge for the near future, to improve epidemiological knowledge and the efficiency of the health care and insurance systems.

## Competing interests

The authors declare that they have no competing interests.

## Authors’ contributions

AM conceived the study and defined its design, participated in the statistical analysis and drafted the manuscript. AS participated in the design of the study, performed the statistical analysis and helped to draft the manuscript. EM participated to conceive the study and its design and helped to draft the manuscript. SI participated to conceive the study and its design and helped to draft the manuscript. All authors read and approved the final manuscript.

## Pre-publication history

The pre-publication history for this paper can be accessed here:

http://www.biomedcentral.com/1471-2458/12/314/prepub
